# Global trends and frontiers in research on exercise training for heart failure: a bibliometric analysis from 2002 to 2022

**DOI:** 10.3389/fcvm.2023.1181600

**Published:** 2023-06-05

**Authors:** Zhen Yang, Zi-Yi Zhong, Cong-Na Zhao, Ming-Yue Yin, Jia-Hui Wang, Jing Wei, Zhi-Shen Wen, Ming Qi

**Affiliations:** ^1^ Faculty of Movement & Rehabilitation Sciences, KU Leuven, Leuven, Belgium; ^2^ School of Biomedical Sciences, University of Leeds, Leeds, United Kingdom; ^3^ Zhaotong Health Vocational College, Zhaotong, China; ^4^ School of Athletic Performance, Shanghai University of Sport, Shanghai, China; ^5^ Cardiac Rehabilitation Center, Fu Wai Hospital of Chinese Academy of Medical Science, Beijing, China; ^6^ Faculty of Movement & Rehabilitation Sciences, KU Leuven, Bruges, Belgium; ^7^ Rehabilitation Medical Centre, People’s Hospital of Ningxia Hui Autonomous Region, Yinchuan, China

**Keywords:** heart failure, exercise training, cardiovascular rehabilitation, bibliometric, knowledge visualization

## Abstract

**Background:**

Heart failure is a common cardiovascular disease that imposes a heavy clinical and economic burden worldwide. Previous research and guidelines have supported exercise training as a safe, effective, and cost-efficient treatment to intervene in heart failure. The aim of this study was to analyze the global published literature in the field of exercise training for heart failure from 2002 to 2022, and to identify hot spots and frontiers within this research field.

**Methods:**

Bibliometric information on literature on the topic of exercise training for heart failure published between 2002 and 2022 was searched and collected in the Web of Science Core Collection. CiteSpace 6.1.R6 (Basic) and VOSviewer (1.6.18) were applied to perform bibliometric and knowledge mapping visualization analyses.

**Results:**

A total of 2017 documents were retrieved, with an upward-stable trend in the field of exercise training for heart failure. The US authors were in the first place with 667 documents (33.07%), followed by Brazilian authors (248, 12.30%) and Italian authors (182, 9.02%). The Universidade de São Paulo in Brazil was the institution with the highest number of publications (130, 6.45%). The top 5 active authors were all from the USA, with Christopher Michael O'Connor and William Erle Kraus publishing the most documents (51, 2.53%). The International Journal of Cardiology (83, 4.12%) and the Journal of Applied Physiology (78, 3.87%) were the two most popular journals, while Cardiac Cardiovascular Systems (983, 48.74%) and Physiology (299, 14.82%) were the two most popular categories. Based on the results of keyword co-occurrence network and co-cited reference network, the hot spots and frontiers of research in the field of exercise training for heart failure were high-intensity interval training, behaviour therapy, heart failure with preserved ejection fraction, and systematic reviews.

**Conclusion:**

The field of exercise training for heart failure has experienced two decades of steady and rapid development, and the findings of this bibliometric analysis provide ideas and references for relevant stakeholders such as subsequent researchers for further exploration.

## 1. Introduction

Heart failure (HF) is a complex clinical syndrome characterized by fatigue and dyspnea due to structural or functional deficiencies in the filling or ejection (1–3). HF affects more than 38 million people worldwide, and it is more prevalent in high-income countries among those aged 65 and older (1, 4). In the latest guidelines, patients are classified as having HF with reduced ejection fraction (HFrEF) (ejection fraction < 40%) or HF with preserved EF (HFpEF) (ejection fraction ≥ 50%). Furthermore, patients with a left ventricular ejection fraction of 40%–49% are classified as having HF with a mid-range ejection fraction (HFmrEF) (1). Patients with HF suffer from exercise intolerance, poor quality of life, and high hospitalization rates, as well as an inferior prognosis than most cancers (4–7). In addition to the substantial clinical burden, HF also imposes a global economic burden of $108 billion per year (8). Consequently, given the significant prevalence, inferior prognosis, and substantial economic burden of HF, it is imperative that stakeholders identify effective, sustainable, and acceptable treatments to optimally enhance the health outcomes of patients with HF.

Taking into account the evidence that cardiac rehabilitation (CR) is effective and safe in improving health outcomes and reducing mortality among patients with HF, the guidelines of the American College of Cardiology/American Heart Association, the European Society of Cardiology, and the Canadian Cardiovascular Society have included exercise training, which is the core of CR, in the treatment of HF (1, 3, 9–14). In spite of its effectiveness, cost-effectiveness, as well as be included in the guidelines, exercise-based CR has not been widely adopted (15–17). Nevertheless, we have witnessed tremendous progress over the past decades in the field of exercise training for heart failure, which is a multidisciplinary field that encompasses from epidemiology to management.

Nakagawa et al. distinguished deep synthesis, like systematic review, as well as broad synthesis, like bibliometric analysis, among the synthesis of both evidence and influence (18). Many deep syntheses, which examining a phenomenon across different trials already exist in the field of exercise training for HF (9, 10, 12). However, there is a lack of broad synthesis on the structure and development of this complex and multidisciplinary field.

Bibliometric analysis is a quantitative statistical tool that combines retrieval and statistics, which is used to describe the knowledge structure and trend hot spots in specific research fields, and can provide comparisons according to the distribution of countries, institutions, authors and periodicals (19, 20), to inform future research. Bibliometric analyses have been extensively used in research field such as HF (21–23), exercise (24–26), and CR (27). Yang et al. conducted a bibliometric analysis on the field of sedentary behavior and cardiovascular disease (28). However, there is a lack of bibliometric analysis in the field of exercise training for HF.

Therefore, this present study aims to conduct a bibliometric analysis of global publication in the field of exercise training for HF to identify (i): publication performance of author, institution, country, journal, and categories, and (ii): research status, hot spots, and frontiers in this field.

## 2. Methods

### 2.1. Data sources

**Figure 1:**
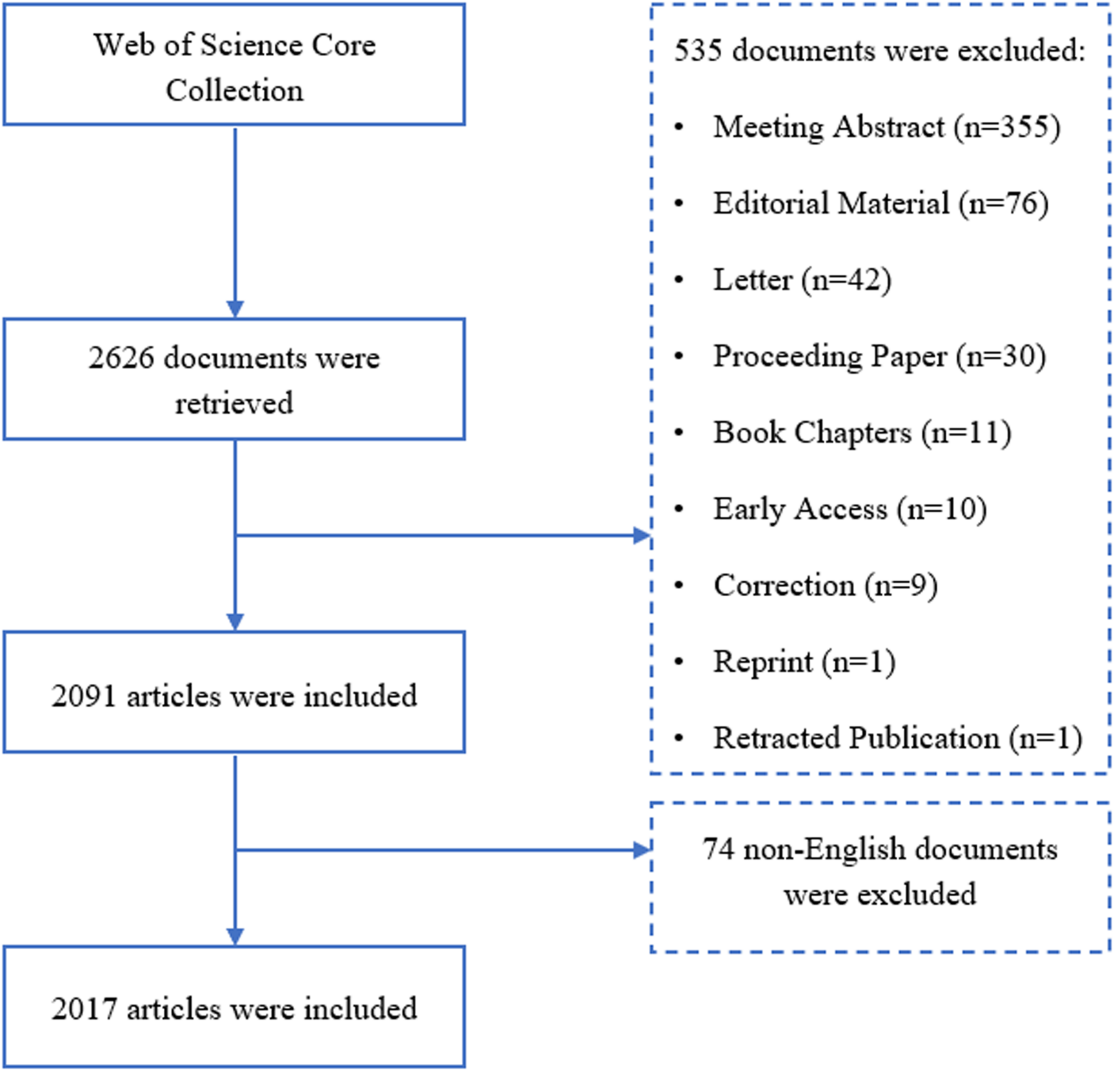
Flow chart of data collection.

This study was conducted according to the bibliometric analysis step-by-step procedure and the best practice guidelines proposed by Donthu et al. (20). There are strong correlations of citation counts across all the disciplines between Web of Science Core Collection (WoSCC), Google Scholar, and Scopus, whereas WoSCC provides earlier documents and more specific, and academic data for bibliometric analysis (29). Meanwhile, other databases of biomedical and life science like PubMed or Scopus, do not provide full text and citation analyses, which are mandatory for bibliometric analysis. Moreover, to eliminate bias caused by the different bibliometric formats of the different databases (30), only the WoSCC was applied for the literature search on the field of exercise training for HF. Following guidelines (20), the search terms in this study were based on a review of the literature of relevant systematic reviews (31, 32), and consultation with experts in the fields of exercise science, public health, physiotherapy, and cardiovascular medicine. The search strategy was as follows: TS = (“exercise training” OR “physical training” OR “exercise therapy”) AND TS = (“heart failure” OR “cardiac failure” OR “ventricular failure” OR “heart decompensation” OR “myocardial failure” OR “heart near failure”). The time span was 2002–2022, and only articles and review articles published in English were included. To eliminate the bias introduced by daily updates of WoSCC, the literature search and data extraction were conducted on 19 December 2022. All data were extracted from WoSCC as plain text files. The procedure of data collection was presented in Figure 1. Initially, the principal investigator conducted a search in the WoSCC database using the search terms described above, hitting 2,626 records. Subsequently, documents that were not articles or review articles were excluded, and 2,091 records were obtained. Finally, after excluding 74 documents that were not published in English, 2017 documents were finally included in the analysis.

### 2.2. Data analysis

Two Java-based bibliometric tools, VOSviewer (1.6.18) and CiteSpace (6.1.R6 Basic), were used to analyze the extracted data. VOSviewer was applied to perform collaborative network analysis of countries/regions, institutions, and authors. CiteSpace was used to conduct keyword co-occurrence analysis, and timeline view of co-cited references. Performance analysis of countries/regions, institutions, authors, journals, categories, and funding agencies was carried out using data automatically generated from the WoSCC.

In CiteSpace, we set the following parameters: Time slicing was from 2002 to 01-01 to 2022-12-19; Year Per Slice was one; Text Processing was Author Keywords; Links were cosine strength within slices; Pruning methods were the pathfinder and the sliced networks; and the Log-Likelihood Ratio was applied as an algorithm for labelling of the clusters generated.

Total link strength and sum of citations were calculated automatically by VOSviewer, and the average number of citations per item (ACI) was derived by dividing the sum of citations by the number of items. The total ‘link strength weighting’ method was applied to demonstrate collaborative networks, in which the size of the node represents the quantity of publications (the larger the diameter the node the more publications). Links represent collaborations between items, with thicker lines representing more collaboration.

## 3. Results

### 3.1. Global publication trends

**Figure 2:**
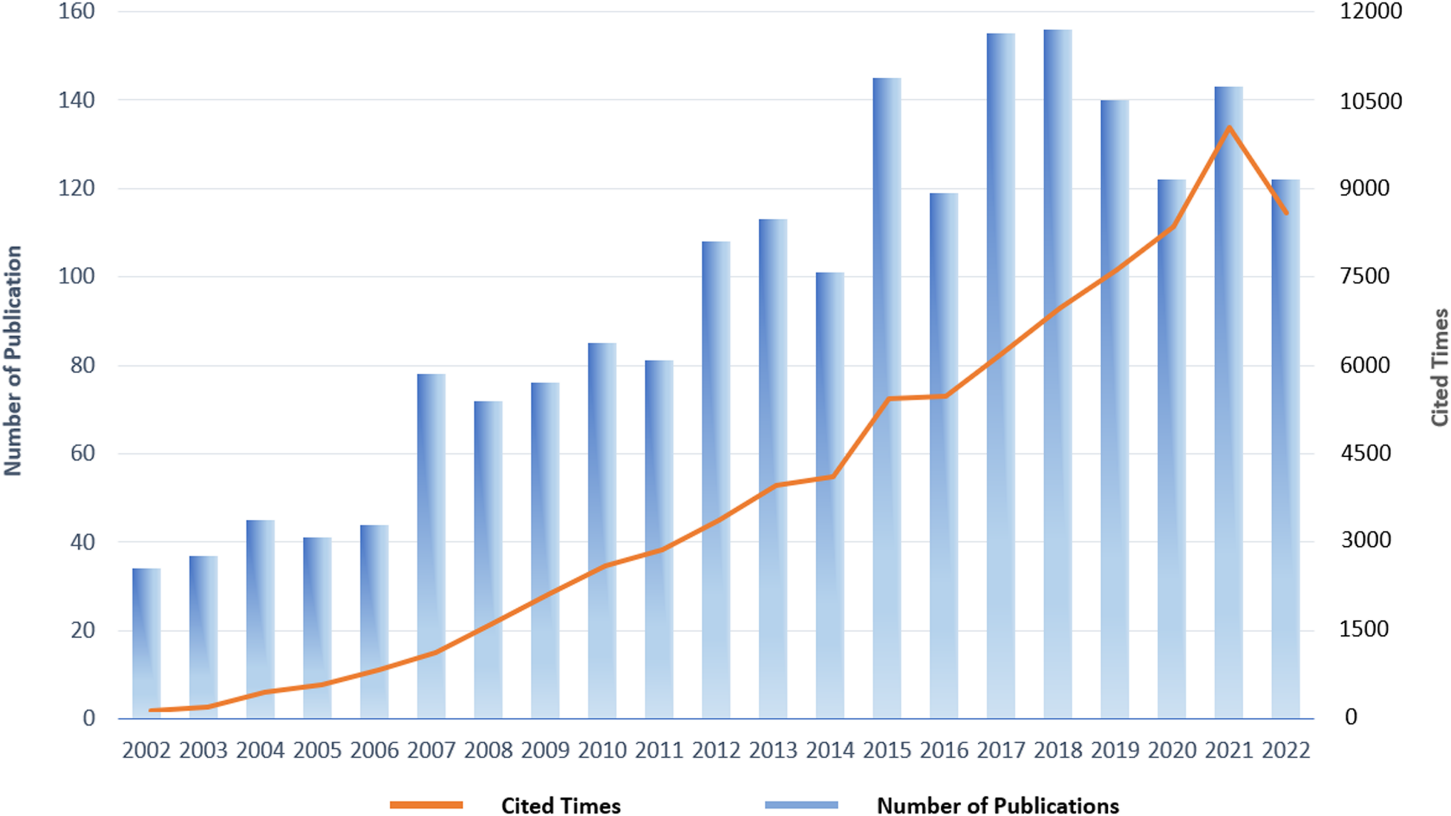
Annual distribution of publications and citations in the field of exercise training for heart failure.

A total of 2017 publications were included in the final analysis, which included 1,515 articles and 502 review articles. The publications included in this study received a total of 80,854 citations, and each publication received an average of 40.09 citations. The annual distribution of publication and citation trends was shown in Figure 2. From 2002 to 2018, there was an increasing publication trend in the field of exercise training for HF until 2018 when it reached the peak of the annual publication (156 documents). Since then, there has been a slight decline, whereas the number of annual publications has maintained stable at over 120 per year. Collectively, the past two decades of publications in the field of exercise training and HF have demonstrated an upward-steady trend and have received continual interest from researchers.

### 3.2. Analysis of countries/regions and institutions

In the field of exercise training for HF, a total of 71 countries/regions and 2,269 institutions contributed to at least one publication between 2002 and 2022. As shown in Table 1, USA had the highest number of publications in the field of exercise training for HF (667 documents), accounting for 33.07% of the total, followed by Brazil, Italy, and Germany. Canada had the highest ACI, followed by Netherlands which represented the high quality of research and academic reputation in the field of exercise training for HF in both countries. Universidade de São Paulo in Brazil contributed the largest number of publications (130 documents). Leipzig University had the highest ACI (108.21), representing its high quality of research and academic reputation in the field of exercise training for HF.

**Table 1:** Top ten active countries/regions and institutions in the field of exercise training for heart failure.

Rank	Country/region	Quantity	%	ACI	TLS	Rank	Institution	Quantity	%	Country/region	ACI	TLS
1	USA	667	33.07	52.19	423	1	Universidade de São Paulo	130	6.45	Brazil	31.46	57
2	Brazil	248	12.30	24.71	105	2	Duke University	95	4.71	USA	70.65	191
3	Italy	182	9.02	51.41	288	3	Henry Ford Hospital	58	2.88	USA	77.62	152
4	Germany	171	8.48	60.11	245	4	The University of Queensland	57	2.83	Australia	54.33	27
5	Australia	158	7.83	57	153	5	Leipzig University	53	2.63	Germany	108.21	27
6	Canada	143	7.09	78.78	159	=6	Thomas Jefferson University	51	2.53	USA	79.91	147
7	England	142	7.04	56.49	257	=6	Norwegian University of Science and Technology	51	2.53	Norway	73.04	78
8	PR China	104	5.16	14.43	41	8	Duke Clinical Research Institute	45	2.23	USA	73.16	130
9	Netherlands	102	5.06	67.04	189	9	University of Alberta	41	2.03	Canada	77.71	31
10	Belgium	90	4.46	48.43	192	10	National Heart, Lung, and Blood Institute	39	1.93	USA	91.74	120

%, Percentage; ACI, average number of citations per item; TLS, Total link strength.

**Figure 3:**
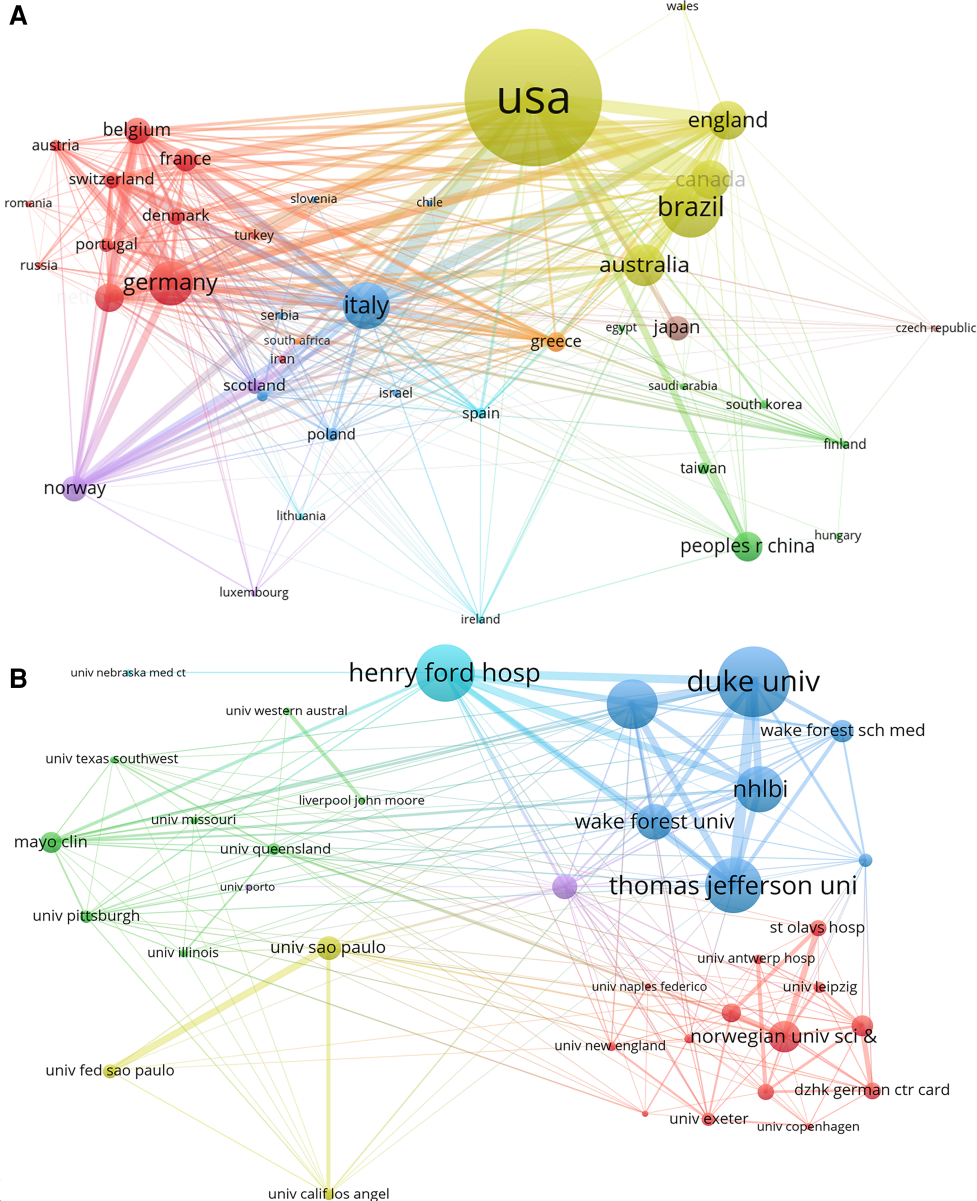
The collaborative network between countries/regions and institutions in the field of exercise training for heart failure. (**A**) Collaborative network map of countries/regions. (**B**) Collaborative network map of institutions.

The number of publications by country/region and institution and their collaborative network were presented by visualization network maps. In VOSviewer, the minimum thresholds were set to 5 and 20 publications for countries/regions and institutions respectively, and a total of 42 countries/regions and 38 institutions reached this threshold and were presented in Figures 3A,B. It is clearly that collaboration was relatively strong across countries, with USA had the strongest collaborative network, followed by Italy, England, and Germany. Among institutions, Duke University had the strongest collaborative network, followed by Henry Ford Hospital and Thomas Jefferson University.

### 3.3. Analysis of authors

**Figure 4:**
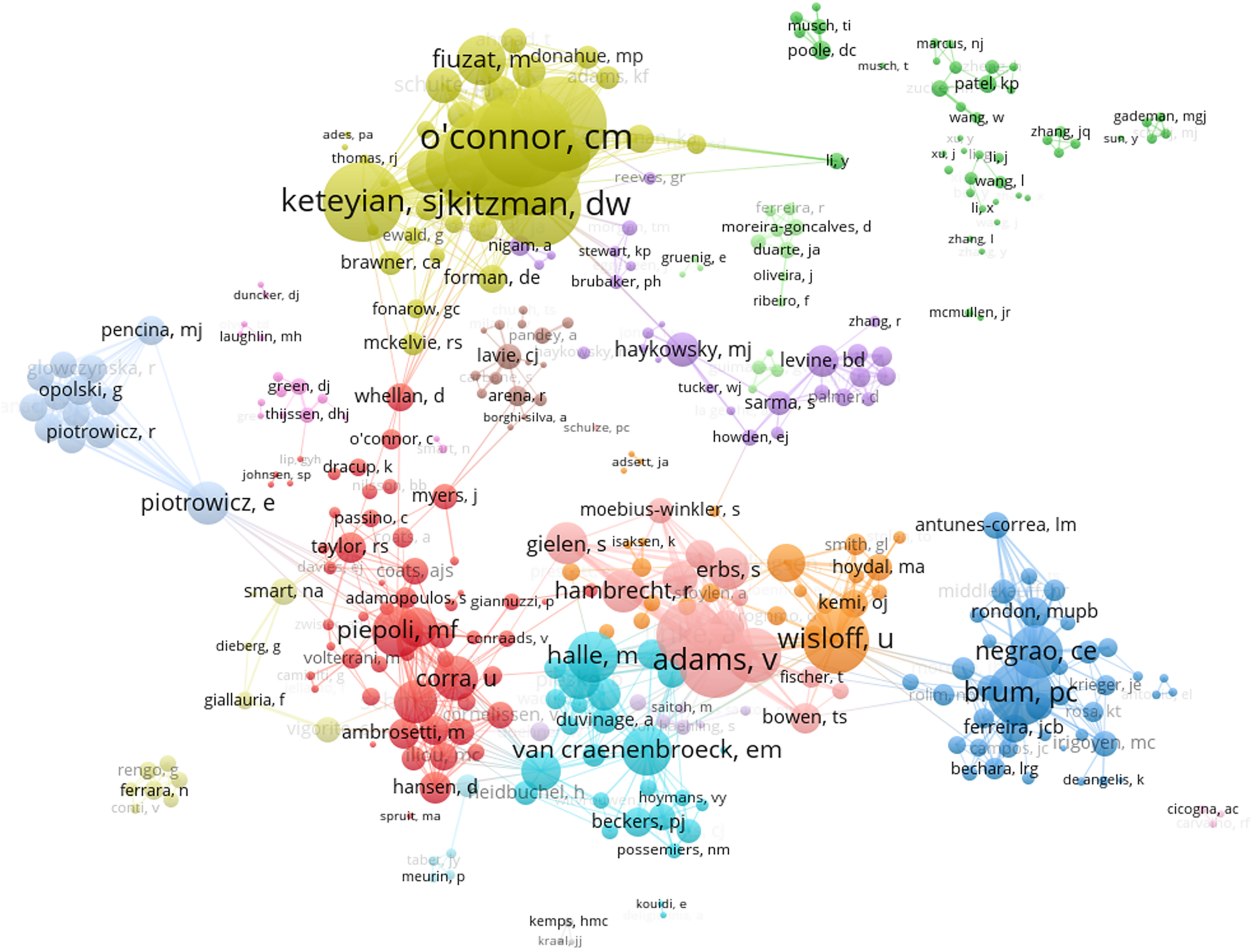
Collaborative network map of authors in the field of exercise training for heart failure.

A total of 8,499 authors contributed to the 2017 publications. Table 2 shows the top ten authors with the highest number of publications in the field of exercise training for HF, seven of whom were from the USA and one from Germany, Norway, and Canada respectively. Christopher Michael O’Connor and William Erle Kraus from Duke University published 51 documents, tied as the most prolific researchers in the field of exercise training for HF. Dalane W. Kitzman from Wake Forest University had the highest ACI (118.5), implying that he may have a high quality of research in this field. In the collaborative networks among authors (Figure 4), the minimum thresholds were set to 5 publications, and a total of 403 authors reached this threshold. Christopher Michael O'Connor had the strongest collaborative network, followed by David J. Whellan and William Erle Kraus.

**Table 2:** Top ten active authors in the field of exercise training for heart failure.

Rank	Author	Institution	Country/region	Quantity	%	ACI	TLS
=1	Christopher Michael O’Connor	Duke University	USA	51	2.53	81.39	355
=1	William Erle Kraus	Duke University	USA	51	2.53	82.49	339
=3	David J. Whellan	Thomas Jefferson University	USA	50	2.48	79.84	346
=3	Dalane W. Kitzman	Wake Forest University	USA	50	2.48	118.5	284
=5	Ileana L Pina	Thomas Jefferson University	USA	46	2.28	87.09	336
=5	Steven J Keteyian	Henry Ford Hospital	USA	46	2.28	81.59	258
7	Adams Volker	Technische Universität Dresden	Germany	42	2.08	52.02	214
8	Ulrik Wisløff	Norwegian University of Science and Technology	Norway	39	1.94	77.08	180
9	Mark Haykowsky	University of Alberta	Canada	32	1.59	65.19	81
10	Jerome L. Fleg	National Heart, Lung, and Blood Institute	USA	29	1.44	97.34	208

%, Percentage; ACI, average number of citations per item; TLS, Total link strength.

### 3.4. Analysis of journals

Documents in the field of exercise training for HF were published in a total of 530 journals, with Table 3 showing the top ten popular journals. Impact factors (IF) and Quartile were reported from the 2021 Journal Citation Report. Three of the top ten popular journals were in quartile 1. The International Journal of Cardiology published the most documents and the JOURNAL OF THE AMERICAN COLLEGE OF CARDIOLOGY had the highest IF (27.206).

**Table 3:** Top ten active journals and co-cited journals in the field of exercise training for heart failure.

Rank	Journal	Quantity	%	IF	Quartile	Rank	Co-cited journal	Citation	IF	Quartile
1	International Journal of Cardiology	83	4.12	4.039	2	1	CIRCULATION	9,718	39.992	1
2	Journal of Applied Physiology	78	3.87	3.881	2	2	JOURNAL OF THE AMERICAN COLLEGE OF CARDIOLOGY	5,882	27.206	1
=3	European Journal of Preventive Cardiology	58	2.88	8.526	1	3	Journal of Applied Physiology	4,062	3.881	2
=3	Journal of Cardiopulmonary Rehabilitation and Prevention	58	2.88	3.697	2	4	EUROPEAN HEART JOURNAL	2,670	35.855	1
5	AMERICAN JOURNAL OF PHYSIOLOGY-HEART AND CIRCULATORY PHYSIOLOGY	48	2.38	5.125	2	5	AMERICAN JOURNAL OF PHYSIOLOGY-HEART AND CIRCULATORY PHYSIOLOGY	2,468	5.125	2
6	EUROPEAN JOURNAL OF HEART FAILURE	43	2.13	18.174	1	6	AMERICAN JOURNAL OF CARDIOLOGY	2,122	3.133	3
7	AMERICAN HEART JOURNAL	41	2.03	5.099	2	7	EUROPEAN JOURNAL OF HEART FAILURE	2,034	18.174	1
8	HEART FAILURE REVIEWS	39	1.93	4.654	2	8	MEDICINE AND SCIENCE IN SPORTS AND EXERCISE	2,026	5.411	1
9	EUROPEAN JOURNAL OF CARDIOVASCULAR PREVENTION & REHABILITATION	36	1.78	3.691	2	9	International Journal of Cardiology	1,958	4.039	2
10	JOURNAL OF THE AMERICAN COLLEGE OF CARDIOLOGY	34	1.69	27.206	1	10	CIRCULATION RESEARCH	1,929	23.218	1

%, Percentage; IF, Impact Factors.

Table 3 shows the top ten popular journals out of a total of 6,149 co-cited journals, four of which were also listed as the top ten journals with the largest number of publications. CIRCULATION was the most cited journal (9,718 documents), followed by JOURNAL OF THE AMERICAN COLLEGE OF CARDIOLOGY (5,882 documents). Of the top ten co-cited journals, six were in Quartile 1, while CIRCULATION had the highest IF (39.992), followed by EUROPEAN HEART JOURNAL (35.855).

### 3.5. Analysis of categories

A total of 77 Web of Science categories were classified for documents in the field of exercise training for HF from 2002–2022. The top ten Web of Science categories in this field is clearly shown in Table 4. Nearly half of the publications were classified as Cardiac Cardiovascular Systems, while Physiology and Sport Science were the second and third most categorized, with over ten percent.

**Table 4:** Top ten Web of science categories in the field of exercise training for heart failure.

Rank	Web of science categories	Quantity	Percentage (%)
1	Cardiac Cardiovascular Systems	983	48.74
2	Physiology	299	14.82
3	Sport Sciences	252	12.49
4	Peripheral Vascular Disease	123	6.10
5	Medicine General Internal	118	5.85
6	Medicine Research Experimental	80	3.97
7	Rehabilitation	73	3.62
8	Respiratory System	70	3.47
9	Cell Biology	67	3.32
10	Pharmacology Pharmacy	59	2.93

### 3.6. Analysis of funding agencies

Among all publications in the field of exercise training for HF from 2002–2022, total of 1,314 research received at least one grant (65.15%). Table 5 presented the top ten funding agencies that provided the largest number of grants, with five from the USA, three from Brazil, and one each from China and Europe. The National Institutes of Health and the United States Department of Health and Human Services from the USA tied for first place, each funded 356 studies.

**Table 5:** Top ten funding agencies in the field of exercise training for heart failure.

Rank	Agency	Quantity	Percentage (%)	Country/region
=1	National Institutes of Health	356	17.65	USA
=1	United States Department of Health and Human Services	356	17.65	USA
3	National Heart, Lung, and Blood Institute	234	11.60	USA
4	National Council for Scientific and Technological Development	122	6.05	Brazil
5	São Paulo Research Foundation	95	4.71	Brazil
6	National Institute on Aging	73	3.62	USA
7	American Heart Association	65	3.22	USA
8	Coordination for the Improvement of Higher Education Personnel	60	2.97	Brazil
9	National Natural Science Foundation of China	54	2.68	China
10	European Commission	53	2.63	Europe

### 3.7. Analysis of keywords

**Figure 5:**
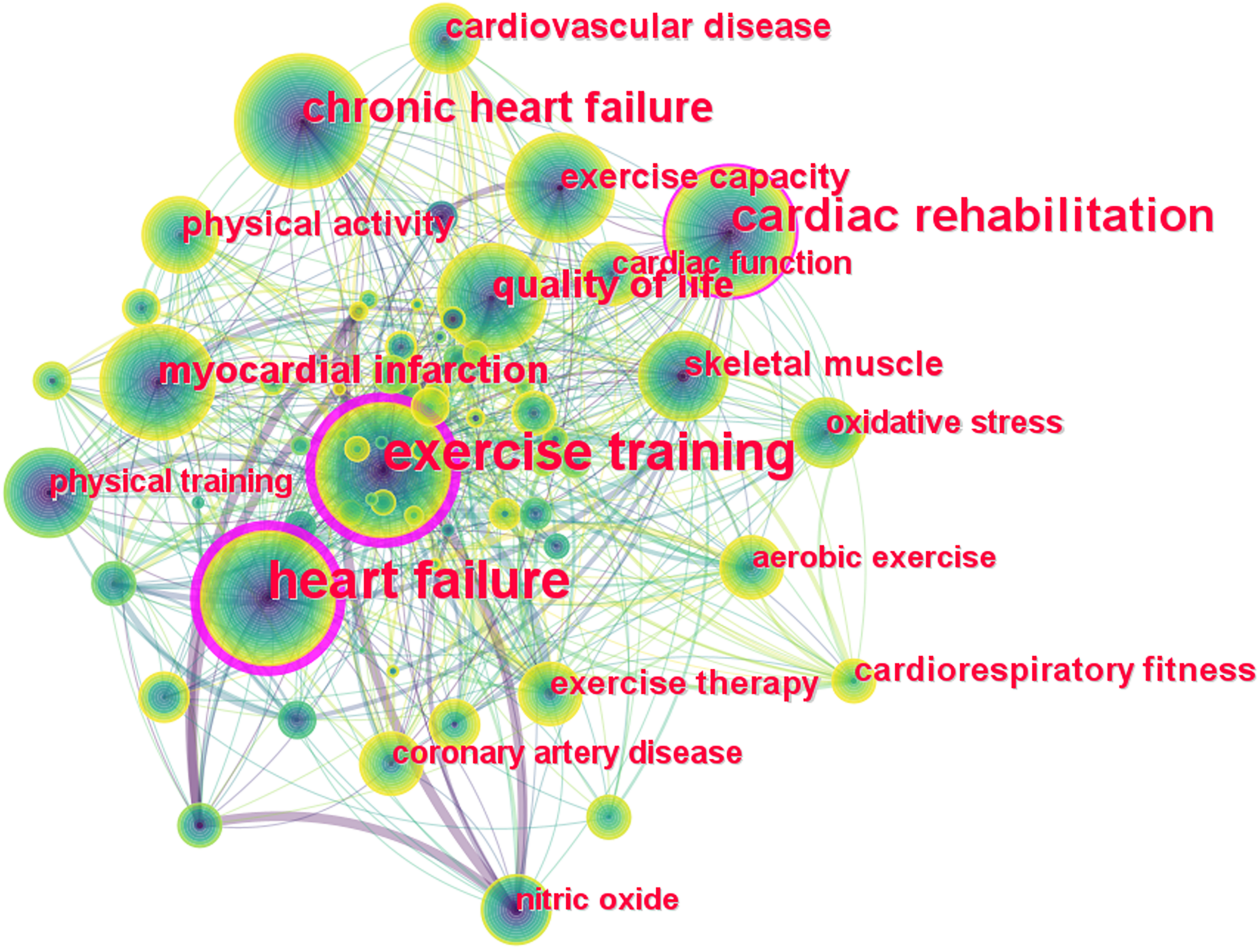
Network map of keyword co-occurrence in the field of exercise training for heart failure.

Keywords are a highly condensed version of the core content and findings in a scientific document, and their number of occurrences and their evolution over time indicate hot spots and directions in a particular field of research. Figure 5 shows the network map of keyword co-occurrence in the field of exercise training for HF, with larger nodes representing more frequent occurrences of the keyword, and thicker links representing more frequent occurrences of both keywords together. The top twenty keywords with the most frequent occurrences are presented in Table 6, which includes types of exercise training, outcome indicators of HF, potential mechanisms: ‘heart failure’ (536), ‘exercise training’ (480), ‘cardiac rehabilitation’ (204), ‘chronic heart failure’ (120), ‘quality of life’ (87), “myocardial infarction” (77) etc.

**Table 6:** Top twenty high-frequency keyword in the field of exercise training for heart failure.

Rank	Keyword	Quantity	Rank	Keyword	Quantity
1	Heart failure	536	=11	Exercise therapy	41
2	exercise training	480	=11	Cardiorespiratory fitness	41
3	Cardiac rehabilitation	204	13	Coronary artery disease	39
4	Chronic heart failure	120	=14	Oxidative stress	38
5	Quality of life	87	=14	Cardiac function	38
6	Myocardial infarction	77	16	physical training	36
7	Physical activity	56	=8	Nitric oxide	31
8	Skeletal muscle	54	=8	Aerobic exercise	30
9	Cardiovascular disease	54	19	blood pressure	25
10	Exercise capacity	52	20	Endothelial function	23

**Figure 6:**
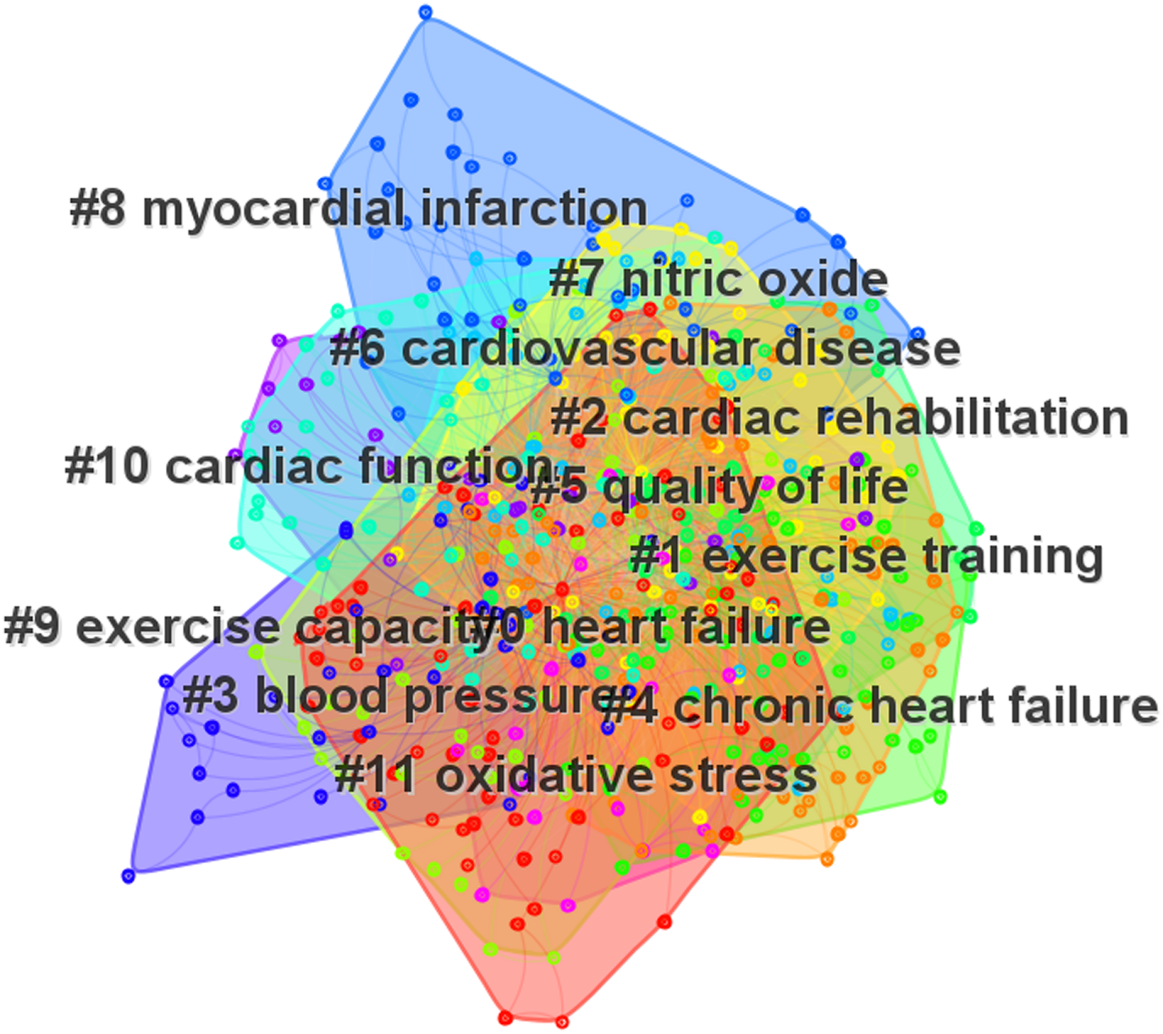
Network map of keyword clusters in the field of exercise training for heart failure.

Keyword clustering contributes to the exploration and analysis of current hot spots and knowledge structures. Figure 6 presents the eleven clusters obtained, excluding #1, #2, #4, #6, #8, which are consistent with the research themes. The remaining clusters were divided into two groups as follows: (i): clusters which related to the outcome measures, including #3 blood pressure, #5 quality of life, #9 exercise capacity, and #10 cardiac function; (ii): clusters which related to the potential mechanism, including #7 nitric oxide and #11 oxidative stress.

**Figure 7:**
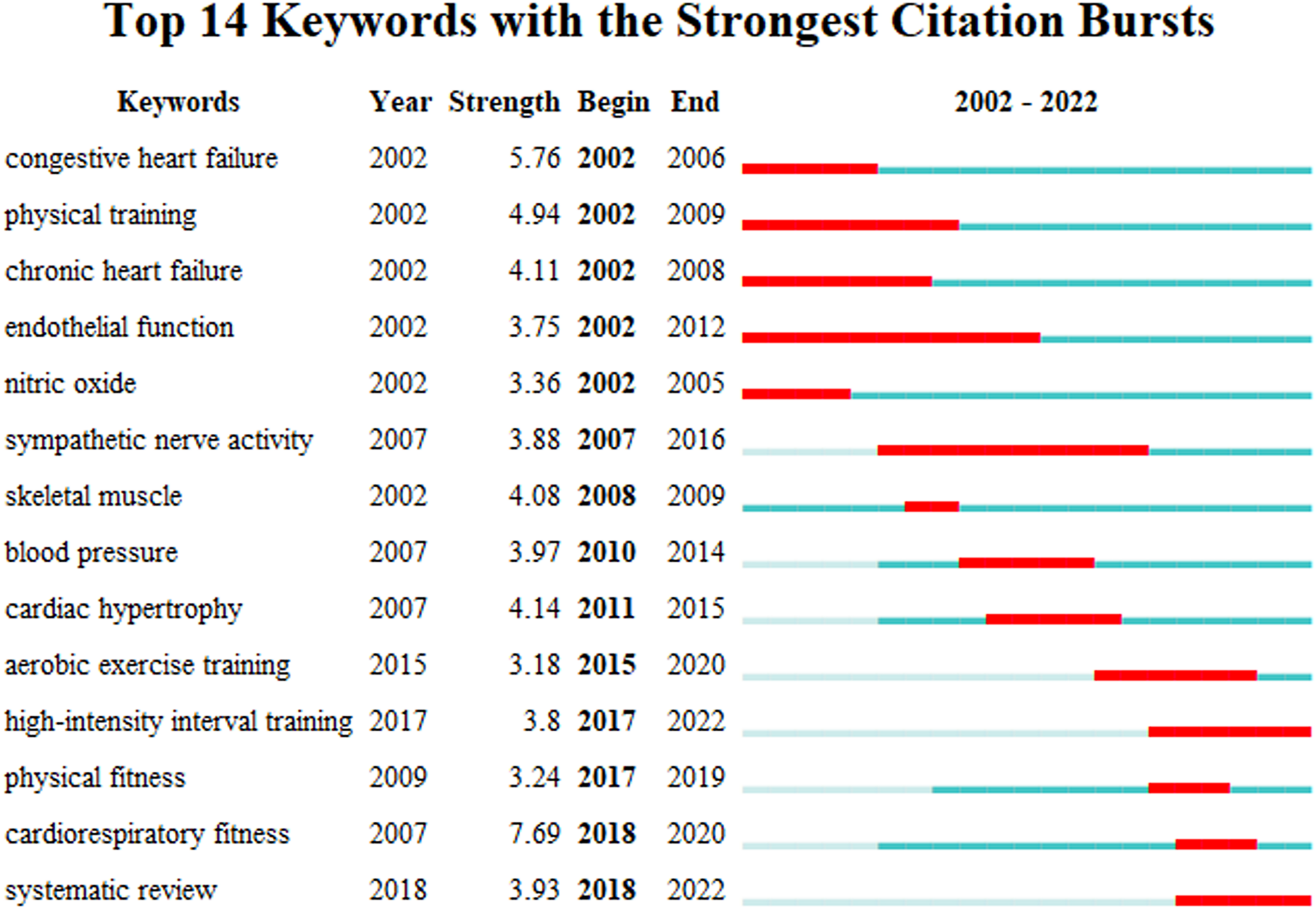
Top fourteen keywords with the strongest citation bursts in the field of exercise training for heart failure.

Keyword citation bursts reflect the trends and frontiers of a research field and contribute to predicting and guiding future research directions. Figure 7 shows the top fourteen keywords with the strongest citation bursts in the field of exercise training for HF, where the red line indicates the duration of a keyword citation burst during 2002–2022 (blue line). The keyword ‘high-intensity interval training’ is continually bursting from 2017 to the present, while the keyword ’systematic review’ is continually bursting from 2018 to the present. This indicates that research on high-intensity interval training (HIIT) and evidence synthesis has continued to receive attention in recent years and may be a trend for future research in the field of exercise training for HF.

### 3.8. Analysis of co-cited reference

A co-cited reference is a document that was cited by both documents, and the analysis of co-cited references contribute to guiding further research hot spots in the field of research. The top ten most co-cited references are presented in Table 7. The document entitled ‘Efficacy and safety of exercise training in patients with chronic heart failure: HF-ACTION randomised controlled trial’ by O’Connor et al. published in JAMA in 2009 was co-cited the most times. This document reported the results of a multicenter, randomized controlled trial designed to investigate the effectiveness and safety of exercise training in patients with chronic HF (33). The second most co-cited document was published in 2016 in the European Heart Journal by Ponikowski et al. entitled: ‘2016 ESC guidelines for the diagnosis and treatment of acute and chronic heart failure: the task force for the diagnosis and treatment of acute and chronic heart failure of the European Society of Cardiology (ESC) developed with the special contribution of the Heart Failure Association (HFA) of the ESC’ (34). This guideline for the diagnosis and treatment of acute and chronic HF recommended regular aerobic exercise to improve functional capacity and symptoms in patients with HF and to reduce the risk of HF hospitalization in stable patients with HFrEF. The document entitled: ’Superior cardiovascular effect of aerobic interval training versus moderate continuous training in heart failure patients: a randomized study’, published by Wisløff et al. in 2007 in CIRCULATION, received the third largest number of co-citations. This randomized controlled trial compared the cardiovascular effects of aerobic interval training with moderate continuous training in patients with HF (35).

**Table 7:** Top ten most co-cited references in the field of exercise training for heart failure.

Rank	Title	First author	Journal	Year	Citation
1	Efficacy and Safety of Exercise Training in Patients With Chronic Heart Failure: HF-ACTION Randomized Controlled Trial	Christopher M. O’Connor	JAMA-JOURNAL OF THE AMERICAN MEDICAL ASSOCIATION	2009	126
2	2016 ESC Guidelines for the diagnosis and treatment of acute and chronic heart failure: The Task Force for the diagnosis and treatment of acute and chronic heart failure of the European Society of Cardiology (ESC)Developed with the special contribution of the Heart Failure Association (HFA) of the ESC	Piotr Ponikowski	EUROPEAN HEART JOURNAL	2016	113
3	Superior cardiovascular effect of aerobic interval training versus moderate continuous training in heart failure patients: a randomized study	Ulrik Wisløff	CIRCULATION	2007	84
4	High-Intensity Interval Training in Patients With Heart Failure With Reduced Ejection Fraction	Øyvind Ellingsen	CIRCULATION	2017	70
5	Effects of exercise training on health status in patients with chronic heart failure: HF-ACTION randomized controlled trial	Kathryn E Flynn	JAMA-JOURNAL OF THE AMERICAN MEDICAL ASSOCIATION	2009	67
6	Exercise-based rehabilitation for heart failure	Rod S Taylor	Cochrane Database of Systematic Reviews	2014	66
7	Exercise training meta-analysis of trials in patients with chronic heart failure (ExTraMATCH)	ExTraMATCH Collaborative	BMJ-British Medical Journal	2004	62
8	2013 ACCF/AHA guideline for the management of heart failure: a report of the American College of Cardiology Foundation/American Heart Association Task Force on Practice Guidelines	Clyde W Yancy	JOURNAL OF THE AMERICAN COLLEGE OF CARDIOLOGY	2013	53
9	Exercise training in heart failure: from theory to practice. A consensus document of the Heart Failure Association and the European Association for Cardiovascular Prevention and Rehabilitation	Massimo F Piepoli	EUROPEAN JOURNAL OF HEART FAILURE	2011	51
10	Exercise and physical activity in the prevention and treatment of atherosclerotic cardiovascular disease: a statement from the Council on Clinical Cardiology (Subcommittee on Exercise, Rehabilitation, and Prevention) and the Council on Nutrition, Physical Activity, and Metabolism (Subcommittee on Physical Activity)	Paul D Thompson	CIRCULATION	2013	50

**Figure 8:**
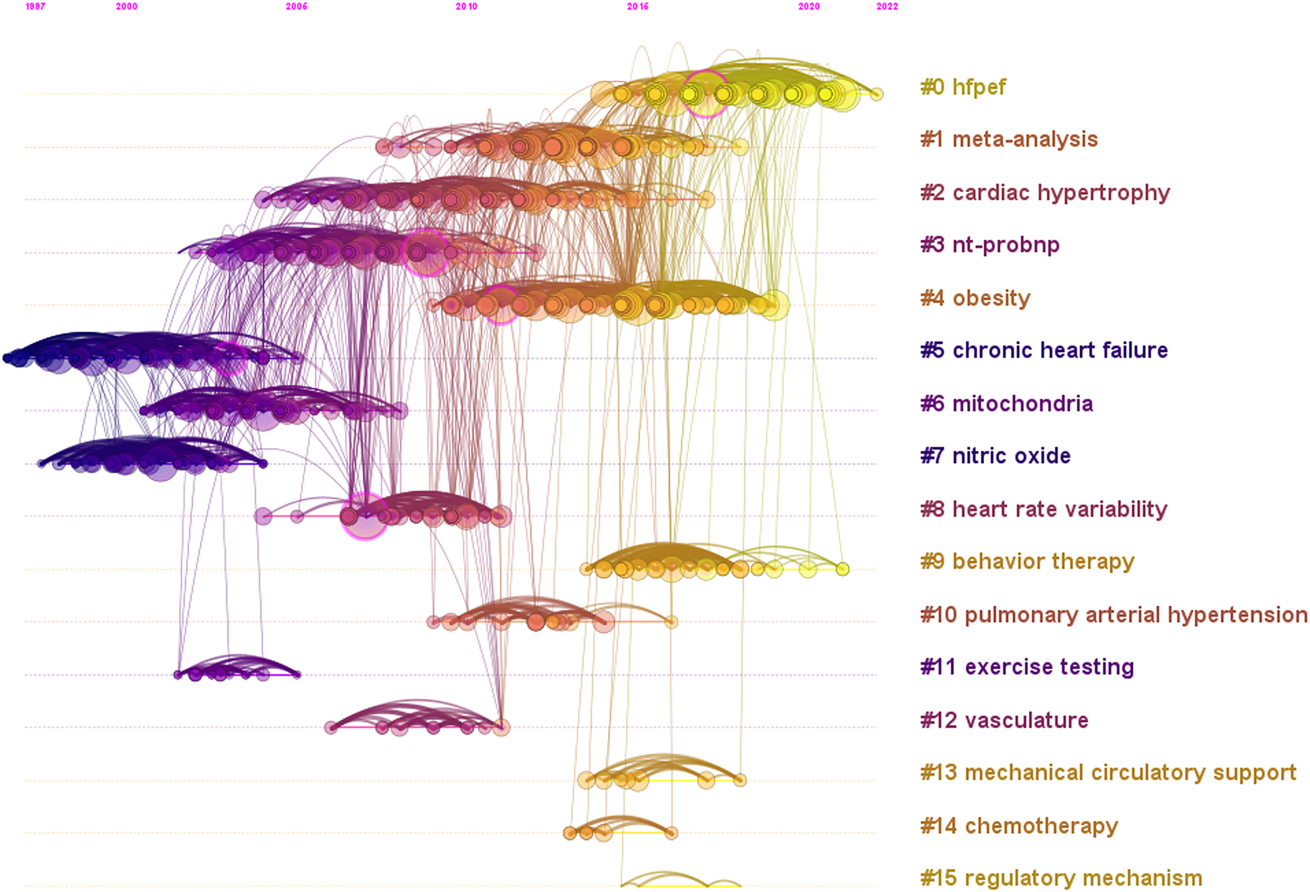
Timeline view of co-cited reference in the field of exercise training for heart failure.

A timeline view of co-cited references in the field of exercise training for HF is presented in Figure 8, indicating a change in research topics over time. Earlier research in this field focused on chronic HF, mitochondria, and nitric oxide. More recently, researchers have focused on HFpEF and behavior therapy, which may be the latest research hot spot and future trend.

## 4. Discussion

### 4.1. General information

Research progress in the field of exercise training for HF can be estimated based on the quantity and trend of annual publications and citations. In general, the number of publications in this field has gradually increased in the past two decades. From 2002–2014, relatively few documents were published, thus relevant research was still in its infancy. Since 2014, publications in this field have entered a period of rapid growth, indicating that research on exercise training for HF has received increasing attention in recent years. The USA contributed the largest quantity of publications and had the strongest collaborative network. In contrast to other active developed countries in this field, such as Italy and Germany, developing countries had a larger number of publications, while the quality of research and cooperation needs to be further enhanced. Among the institutions, while Universidade de São Paulo from Brazil published the largest number of documents, those from the USA, Canada and Germany had higher quality of research. In terms of institutional collaboration, only the USA institutions had strong collaborative networks. Overall, apart from the USA, cooperation between countries and institutions were weak, thus strengthening international and institutional cooperation, especially in developing countries, is a priority in this field. The scientists with the largest number of publications, led by Christopher M O’Connor, William E Kraus, David J Whellan, and Dalane W Kitzman, are the backbone of scientific productivity in the field of exercise training for HF and are all from the USA. Through analysis of journals, this study found that the top ten most productive journals in the field of exercise training for HF are all quartile 1 or quartile 2 journals. Therefore, it is achievable to publish research on the topic of exercise training for HF on high quality journals.

### 4.2. Hot spots and frontiers

Based on the citation burst of co-occurring keywords and the timeline view of co-cited reference, we identified the following hot spots and frontiers in the field of exercise training for HF. HIIT is the promising and cutting-edge exercise training treatment in this field. HIIT is defined as repeated high-intensity interval bouts between 80% and 100% of peak heart rate interspersed with recovery periods or light exercise (36, 37). It is initially designed to improve the aerobic capacity of runners, was first adopted as a treatment for patients with coronary artery disease and chronic HF in 1990 (38, 39). This emerging exercise training method can further enhance the metabolic, cardiopulmonary, and systemic vascular adaptations of patients with HF (36), and has been demonstrated to be safe for patients with cardiac pathology (40). This type of exercise training is often used to compare with traditional moderate intensity training. Recent meta-analyses proposed that HIIT is significantly more effective than moderate intensity training for improving peak VO_2_ value and left-ventricular ejection fraction among patients with HF, which are independent predictor of cardiovascular mortality in patients with cardiovascular diseases (41, 42). Furthermore, a previous meta-analysis recommended that patients with HF conduct HIIT at least three days a week, while the active recovery intervals should be between 40% and 60% of the peak VO_2_ (43).

HFpEF is a active type of HF in the field of exercise training for HF. This type of HF is a condition with similar clinical manifestations and event rate to HFrEF (44, 45), but different in etiology, cardiac remodeling, pathophysiology, comorbid disorders, and response to therapy (44). CR for patients with HFpEF has recently received increasing attention. A recent systematic review with meta-analysis examined reported that exercise training significantly improved cardiorespiratory fitness, with no difference in peak VO_2_ between resistance and aerobic exercise, while HIIT significantly improved peak VO_2_ compared to aerobic exercise (46). Another meta-analysis including the largest sample size to date in this field proposed that exercise training in patients with HFpEF improved their exercise tolerance and somatic-related quality of life, but not emotional or mental quality of life or echocardiographic parameters (47). It is noted that the majority of clinical trials included in recent systematic reviews on exercise training and HFpEF were small sample-sized randomized controlled trials rather than large multicenter trials, which may have reduced the strength of evidence for the findings. Therefore, more robustly designed, multicenter large randomized clinical trials are needed in the future to further examine the effects of different types of exercise training on relevant indicators in patients with HFpEF.

Systematic review is a hotly mentioned study design in the field of exercise training for HF and is at the heart of evidence-based medicine (48). The American Heart Association considered the evidence from systematic reviews and meta-analyses to be ‘A’ level, assuring the highest confidence. However, Murad et al. suggest using this as a lens to observe other types of studies, that is, evaluations and applications, given the different risks of bias in different systematic reviews (49). Furthermore, systematic review with meta-analysis is tools for stakeholders to access and apply evidence (49). Researchers can identify research gaps and future research orientations based on the evidence derived from systematic reviews. Policy makers can use the Grading of Recommendations Assessment, Development and Evaluation to determine the strength of evidence from systematic reviews and to develop relevant policies and guidelines (50). Clinical practitioners can be guided in their clinical practice by evidence-based guidelines that include evidence from systematic reviews. In the field of HF management, national and international evidence-based guidelines have incorporated exercise training into treatment and management (3, 34, 51). However, implementation is limited by the need to safely select patients and develop exercise prescriptions through cardiopulmonary exercise testing and the lack of coverage by the Centers for Medicare and Medicaid Service for patients with HFpEF (52, 53).

### 4.3. Limitation

This research is the first bibliometric analysis of exercise training for HF, but there are some inevitable limitations. First, this study only included literature published in English, and there may have been some omissions for studies published in other languages. However, given the impact of English-language publications, the impact of this exclude criteria was limited. Second, this study only searched one electronic database, WOSCC, and may have omitted publication in other databases and grey literature. This is because different databases have different rules for abbreviating author names, etc., and integrating data from different databases may result in a significant amount of duplication. Furthermore, only articles and review articles were included in the analysis, while WoSCC has more accurate document type labelling than alternative database such as Scopus (54).

## 5. Conclusion

This study applied state-of-the-art bibliometric and scientific mapping methods to provide researchers and other stakeholders with a panoramic view of the field of exercise training for HF from 2002–2022. Publications in the field show an upward-stable trend, with developed countries, led by the USA, maintaining a high number and quality of publications at the same time. Brazil and China represent the developing countries in this field, but there is a need to improve the quality of research as well as the international cooperation. In the field of exercise training for HF, the hot and cutting-edge type of exercise training is HIIT, while HFpEF is the type of heart failure that has received attention, and systematic review is the promising study design.

## Data Availability

The raw data supporting the conclusions of this article will be made available by the authors, without undue reservation.
